# Associations between high birth weight, being large for gestational age, and high blood pressure among adolescents: a cross-sectional study

**DOI:** 10.1007/s00394-016-1372-0

**Published:** 2017-01-05

**Authors:** Renata Kuciene, Virginija Dulskiene, Jurate Medzioniene

**Affiliations:** Laboratory of Population Studies, Institute of Cardiology, Medical Academy, Lithuanian University of Health Sciences, Sukileliu 15, 50009 Kaunas, Lithuania

**Keywords:** High blood pressure, High birth weight, Large for gestational age, Overweight, Obesity, Adolescents

## Abstract

**Purpose:**

Low birth weight and being small for gestational age are associated with increased risk of cardiometabolic diseases. However, the results from the studies examining the associations between high birth weight (HBW), being large for gestational age (LGA), and high blood pressure (HBP) are inconsistent. The aim of this study was to evaluate the associations between HBW and being LGA alone and in combinations with body mass index (BMI) categories in adolescence and HBP among Lithuanian adolescents aged 12–15 years.

**Methods:**

The participants with HBP (≥90th percentile) were screened on two separate occasions. Data on the BMI, birth weight (BW), gestational age, and BP were analyzed in 4598 adolescents. Adjusted odds ratios (aORs) with 95% confidence intervals (CI) for the associations were estimated using multivariate logistic regression models.

**Results:**

The overall prevalence of HBW (>4000 g), being LGA, adolescent overweight/obesity, and HBP were 13.9, 10.4, 14.5, and 25.6%, respectively. After adjustment for age, sex, and BMI, significant positive associations were found between HBW and being LGA and HBP (HBW: aOR 1.34; 95% CI, 1.11–1.63; LGA: aOR 1.44; 95% CI, 1.16–1.79). After adjustment for age and sex and compared to BW 2500–4000 g and being AGA (appropriate for gestational age) with normal weight in adolescence, the combinations that included both risk factors—HBW with overweight/obesity and being LGA with overweight/obesity—showed higher aORs (aOR 4.36; 95% CI, 3.04–6.26; and aOR 5.03; 95% CI, 3.33–7.60, respectively) than those with either of these risk factors alone did.

**Conclusions:**

HBW and being LGA were positively associated with HBP in Lithuanian adolescents aged 12–15 years. The highest odds of having HBP were observed for subjects with both risk factors—neonatal HBW or being LGA and overweight/obesity in adolescence.

## Introduction

High blood pressure is a serious, growing, and global public health problem [1]. It is the leading risk factor for cardiovascular and circulatory diseases (e.g., ischaemic heart disease, rheumatic heart disease, ischaemic stroke, haemorrhagic and other nonischaemic stroke, and hypertensive heart disease) [2]. According to the World Health Organization, HBP causes 7.5 million deaths per year worldwide (about 13% of all deaths) [3]. A systematic review and a meta-regression analysis including 55 studies with a total of 122,053 adolescents have showed that the pooled prevalence of HBP was 13% for boys and 9.6% for girls [4]. It has been demonstrated that BP tracks significantly from childhood to adulthood [5]. Research literature suggests that HBP can be influenced by environmental factors and genetic factors as well as interactions between these factors [6].

It has also been established that obesity is associated with an increased risk of cardiovascular and other non-communicable diseases among children, adolescents, and adults [3, 7]. In Europe, the prevalence of obesity is increasing in the general population, including women of reproductive age and children born with HBW. Women who are obese (particularly with a pre-pregnancy metabolic syndrome or gestational diabetes) are at an increased risk for adverse neonatal outcomes such as fetal macrosomia and LGA neonates [7]. Scientific studies report that HBW is associated with a higher risk of obesity in childhood and adulthood, and can influence the development of cardiovascular diseases [8].

The increasing scientific evidence of the associations between HBW and hypertension or HBP has been described and summarized in several systematic reviews and meta-analyses [9, 10]. For example, Mu et al. found that there is an inversely linear association between BW and the risk of adult hypertension [9]. Zhang et al. reported that HBW (BW ≥4000 g or ≥90th percentile for the gestational age) was related to a higher risk of HBP and hypertension in younger children, but with a lower risk in older adults, compared to those with normal birth weight (BW 2500–4000 g or the 10th–90th percentiles for gestational age) [10]. Even though epidemiological studies have examined the association between HBW and HBP [11–13] or hypertension [14, 15] among children and adolescents, the results have been inconsistent.

The relationships between HBP and HBW among adolescents have not been studied in Lithuania before. In Lithuania, epidemiological studies indicated a high prevalence of HBP in children [16], adolescents [17], and adults [18]. The data of the Health Statistics of Lithuania showed that mortality from cardiovascular diseases in Lithuania still remains one of the highest in Europe [19]. In 2014, more than one-half of all deaths (56.0%) in our country were caused by cardiovascular diseases [19]. It is, therefore, essential to identify the risk factors associated with the development of cardiovascular diseases and other chronic non-communicable diseases, and then to make every effort to prevent and control these factors with a particular focus on children, adolescents, and youth. An early detection and modification of the risk factors at a young age may ensure better health and improved quality of life, decreased morbidity or mortality, and healthy aging.

The aim of this study was to evaluate the associations between HBW and being LGA alone and in combination with BMI categories and HBP in Lithuanian adolescents aged 12–15 years. We hypothesized that HBW and being LGA could be positively associated with HBP in adolescence and that subjects with HBW and LGA at birth with overweight/obesity in adolescence might have higher odds of HBP, compared to those with other combinations of either of the risk factors alone.

## Methods

### Study population

For this data analysis, we selected data of 5530 residents of Kaunas city (the second largest city of Lithuania) aged 12–15 years, who participated and were examined in our cross-sectional study that was performed in Kaunas city and Kaunas district and was presented in previous publications [17, 20]. A cross-sectional study included children and adolescents aged 12–15 years who at the time of the examination (from November 2010 to April 2012) attended gymnasiums or secondary schools of Kaunas city, located in Kaunas County, Lithuania. All the invited schools (*n* = 56) accepted the invitation to participate in the research project.

A two-stage sampling design was used to produce a sample of schoolchildren (grades 6–9; ages 12–15 years) of Kaunas city gymnasiums or secondary schools. Stage one of the sampling included all the above-mentioned schools of Kaunas city with schoolchildren aged 12–15 years. Stage two consisted of the sampling of all classes (grades 6, 7, 8, and 9) of all the participating schools. All 12–15-year-old school children from these selected classes were included in the survey.

A total of 97 subjects were excluded from the statistical analyses because they had any of the following conditions: endocrine diseases, kidney diseases, cardiovascular diseases, and congenital heart defects [the information was collected from the subjects’ medical records (Form No. 027-1/a)]. In addition, 16 subjects were excluded due to missing data on anthropometric measurements. We also excluded 490 subjects with missing data on birth weight and gestational age. For the present study, information on BW and gestational age was collected from the medical records of the participants born in Kaunas city during 1995–1998. Multiple births (i.e., twins, triplets, etc.) (*n* = 108) were excluded from the analysis. In addition, cases of premature singleton birth (gestational age <37 weeks) and low birth weight (<2500 g) were excluded from the analysis as well (*n* = 221). Thus, finally, data from 4598 singleton subjects were approved for current statistical analysis.

Both BP and anthropometric measurements were performed at the participants’ schools by the same team of trained study personnel (physicians and research assistants). A written informed consent was obtained from each participant’s parent or guardian. The study was approved by Kaunas Regional Ethics Committee for Biomedical Research at the Lithuanian University of Health Sciences (protocol No. BE-2-69).

### Blood pressure measurements

Blood pressure was measured in the morning hours (8:30–11:30 am) by a physician who was not wearing a white coat. The subjects were advised to avoid tea, coffee, energy drinks, and physical exercises in the morning of the examination day until the measurements were taken. Before the BP measurement, the participants were asked to sit still for 10 min. BP was measured three times with a 5-min rest interval between the measurements, with the participant being in a sitting position; BP was measured using an automatic BP monitor (OMRON M6; OMRON HEALTHCARE CO., LTD, Kyoto, Japan). The average of three BP measurements was calculated. All subjects with an increased BP (BP was in the ≥90th percentile) during the first screening underwent a second evaluation of BP measurements within the period of 2–3 weeks.

Classifications of BP levels and definitions were used according to “The Fourth Report on the Diagnosis, Evaluation and Treatment of High Blood Pressure in Children and Adolescents” [National High Blood Pressure Education Program (NHBPEP) Working Group on High Blood Pressure in Children and Adolescents] [21]. According to BP charts for age, sex, and height, normal BP was defined as systolic blood pressure (SBP) and diastolic blood pressure (DBP) <90th percentile; HBP was defined as average SBP or DBP levels ≥90th percentile.

### Anthropometric measurements

Weight and height of the participants (wearing only light clothing and barefooted) were measured using a portable stadiometer and a balance beam scale (SECA measuring equipment). Weight was measured to the nearest 0.1 kg, and height was measured to the nearest 0.1 cm. The body mass index (BMI) was calculated as weight in kilograms divided by the square of height in meters. Waist circumference (WC) was measured with a flexible measuring tape (SECA) at a level midway between the lower rib margin and the iliac crest. WC was measured to the nearest 0.5 cm.

According to the age- and sex-specific cutoff points of BMI proposed by the International Obesity Task Force [22], the subjects were grouped into the following categories of BMI: normal weight, overweight, and obese.

Data on birth weight and gestational age were abstracted from the medical records. Normal BW was defined as infant’s BW between ≥2500 and ≤4000 g, while HBW was defined as BW >4000 g [23].

Low birth weight was defined as birth weight of <2500 g, and premature birth was defined as birth at <37 weeks of gestation, according to Codes P07.0-3, International Statistical Classification of Diseases and Related Health Problems, 10th revision [24].

According to sex-specific birthweight percentiles by gestational age based on national data in Lithuania [25], newborns were divided into three groups: small for gestational age (SGA) neonates (BW <10th percentile), appropriate for gestational age (AGA) neonates (BW ≥10th–≤90th percentile); large for gestational age (LGA) neonates (BW >90th percentile). Only singleton births were included in our analysis. Preterm infants (gestational age <37 weeks) and low birth weight neonates (<2500 g) were excluded from the analysis.

### Statistical analysis

Categorical variables were expressed as numbers (*n*) and percentages (*%*), and were compared using the Chi-square (*χ* ^2^) test. Means and standard deviations (SD) were presented for the normally distributed continuous variables. The normality of the distribution of the continuous variables was tested by applying the Kolmogorov–Smirnov test. The *t* test was used to compare the mean values of normally distributed variables across the groups.

Univariate and multivariate logistic regression analyses were conducted for both sexes combined to evaluate the associations between HBW, being LGA, and the combinations of HBW and being LGA with different status of BMI in adolescence and HBP. Crude odds ratios (OR) and adjusted odds ratios (aOR) along with 95% confidence intervals (CI) were calculated. In the multivariate analysis, two models were used (for the associations of HBW and being LGA with HBP): in the first model, ORs were adjusted for age and sex; in the second model, ORs were adjusted for age, sex, and BMI. The age- and sex-adjusted models were used to determine the associations of the combinations of anthropometric factors with HBP.

Statistical analyses were performed using the statistical software package SPSS version 20 for Windows. *P* values <0.05 were considered to be statistically significant.

## Results

The mean values of demographic, anthropometric, and BP data for the whole sample and for each sex separately are given in Table 1. Boys had a significantly higher mean BMI, BW, and WC, and they had a significantly higher mean SBP and a significantly lower mean DBP, compared to girls. There were no significant differences in age and gestational age when comparing both sexes.

**Table 1:** Demographic, anthropometric, and BP characteristics of the study participants by sex

Variables	Total (*n* = 4598)	Boys (*n* = 2103)	Girls (*n* = 2495)	P*
Age (years)	13.60 ± 1.04	13.60 ± 1.05	13.61 ± 1.03	0.851
Birth weight (g)	3549.29 ± 442.59	3621.71 ± 454.16	3488.25 ± 423.17	<0.001
Gestational age (weeks)	39.71 ± 0.99	39.68 ± 1.01	39.73 ± 0.98	0.119
Height (cm)	165.78 ± 9.53	167.99 ± 11.08	163.92 ± 7.52	<0.001
Weight (kg)	54.56 ± 14.63	56.78 ± 18.19	52.69 ± 10.39	<0.001
BMI (kg/m^2^)	19.72 ± 5.47	19.96 ± 7.31	19.52 ± 3.16	<0.001
WC (cm)	67.25 ± 7.73	69.53 ± 7.98	65.32 ± 6.96	<0.001
SBP (mmHg)	118.38 ± 14.21	121.96 ± 15.91	115.36 ± 11.78	<0.001
DBP (mmHg)	65.87 ± 7.74	65.40 ± 7.80	66.27 ± 7.67	<0.001

Values are presented as mean ± SD

*BP* blood pressure, *BMI* body mass index, *WC* waist circumference, *SBP* systolic blood pressure, *DBP* diastolic blood pressure

*Boys versus girls

Table 2 presents the characteristics of the participants according to the BP level. Overall, the prevalence of HBP was 25.6% (33.8% for boys and 18.7% for girls). Girls were more frequently normotensive than boys were (81.3 versus 66.2%). Older subjects (aged 14–15 years) were significantly more likely to have HBP than younger subjects (aged 12–13 years) did (29.5 versus 21.1%). The overall prevalence of overweight/obesity was 14.5% (17.3% for boys and 12.2% for girls). The overall prevalence of HBW and being LGA was 13.9% (18.2% for boys and 10.3% for girls) and 10.4% (10.8% for boys and 10.0% for girls), respectively. The risk factors (HBW, being LGA, and overweight/obesity during adolescence, and the combinations of neonatal BW with adolescent BMI categories, including at least one or both of the above-mentioned risk factors) were more prevalent among participants with HBP than among normotensives. The participants with HBP had significantly higher mean values for age, BW, weight, height, BMI, WC, SBP, and DBP, compared to participants with NBP (Table 2).

**Table 2:** Characteristics of the study participants according to the BP level

Variables	NBP (*n* = 3420)		HBP (*n* = 1178)		*P* value
	*n*	*%*	*n*	*%*	
Sex					
Boys	1392	40.7	711	60.4	<0.001
Girls	2028	59.3	467	39.6	
Age (years)					
12–13	1676	49.0	449	38.1	<0.001
14–15	1744	51.0	729	61.9	
BMI categories					
Normal weight	3082	90.1	848	72.0	<0.001
Overweight	298	8.7	255	21.6	
Obesity	40	1.2	75	6.4	
BMI categories					
Normal weight	3082	90.1	848	72.0	<0.001
Overweight/obesity	338	9.9	330	28.0	
Birth weight categories (g)					
2500–4000	2996	87.6	962	81.7	<0.001
>4000	424	12.4	216	18.3	
Birth weight for gestational age					
AGA	2830	82.7	927	78.7	<0.001
SGA	272	8.0	92	7.8	
LGA	318	9.3	159	13.5	
Birth weight (g) and BMI categories					
BW 2500–4000 and normal weight	2718	79.5	705	59.8	<0.001
BW >4000 and normal weight	364	10.6	143	12.2	
BW 2500–4000 and overweight/obesity	278	8.1	257	21.8	
BW >4000 and overweight/obesity	60	1.8	73	6.2	
Birth weight for gestational age and BMI categories					
AGA and normal weight	2551	74.6	678	57.5	<0.001
SGA and normal weight	257	7.5	69	5.9	
LGA and normal weight	274	8.0	101	8.6	
AGA and overweight/obesity	279	8.2	249	21.1	
SGA and overweight/obesity	15	0.4	23	2.0	
LGA and overweight/obesity	44	1.3	58	4.9	
Age (years)	13.54 ± 1.04		13.78 ± 1.02		<0.001
Birth weight (g)	3533.40 ± 437.12		3595.41 ± 455.17		0.001
Gestational age (weeks)	39.73 ± 0.98		39.64 ± 1.02		0.006
Weight (kg)	52.05 ± 10.59		61.86 ± 20.95		<0.001
Height (cm)	164.55 ± 9.19		169.37 ± 9.62		<0.001
BMI (kg/m^2^)	19.09 ± 2.83		21.54 ± 9.44		<0.001
WC (cm)	65.91 ± 6.81		71.14 ± 8.87		<0.001
SBP (mmHg)	111.70 ± 7.54		137.75 ± 10.86		<0.001
DBP (mmHg)	63.85 ± 6.42		71.74 ± 8.22		<0.001

Values are numbers (percentages) and mean ± SD (standard deviation)

*NBP* normal blood pressure, *HBP* high blood pressure, *BMI* body mass index, *BW* birth weight, *WC* waist circumference, *SBP* systolic blood pressure, *DBP* diastolic blood pressure, *SGA* small for gestational age, *AGA* appropriate for gestational age, *LGA* large for gestational age, *SD* standard deviation

The univariate analysis revealed that HBW and being LGA were significantly associated with higher odds of HBP (Table 3) compared to the participants with BW 2500–4000 g and AGA. In the multivariate analysis, adjustments for age and sex in the first models did not affect the significance of the associations of HBW and being LGA with HBP; even though the aORs changed slightly, but they remained statistically significant (Table 3). According to the second model (adjustment for age, sex, and BMI), HBW (>4000 g) and being LGA were also significantly associated with higher odds for HBP (aOR 1.34 and aOR 1.44, respectively), compared to the reference groups.

**Table 3:** Associations between birth weight and high blood pressure (univariate and multivariate analyses)

Variables	OR (95% CI) *P* value	aOR^1^ (95% CI) *P* value	aOR^2^ (95% CI) *P* value
Birth weight categories (g)			
2500–4000	1.00	1.00	1.00
>4000	1.59 (1.33–1.90) *P* < 0.001	1.43 (1.19–1.72) *P* < 0.001	1.34 (1.11–1.63) *P* = 0.002
Birth weight for gestational age			
AGA	1.00	1.00	1.00
LGA	1.53 (1.24–1.87) *P* < 0.001	1.54 (1.25–1.90) *P* < 0.001	1.44 (1.16–1.79) *P* = 0.001

*OR* crude odds ratio, *aOR* ^*1*^ adjusted odds ratios for age and sex, *aOR* ^*2*^ adjusted odds ratios for age, sex, and the body mass index, *CI* confidence interval, *AGA* appropriate for gestational age, *LGA* large for gestational age

Further univariate and multivariate logistic regression analyses regarding the associations of the combinations of the categories of anthropometric parameters (neonatal BW with BMI in adolescence) in relation to the odds of having HBP were conducted (Table 4). In the multivariate analysis, after adjusting for age and sex, the participants in whom these combinations included either or both of the risk factors (HBW and/or overweight/obesity) had significantly higher aORs for HBP, compared to subjects with normal BW and normal weight status in adolescence. When birth weight for gestational age and BMI categories were combined, the participants with the combination of risk factors (LGA with normal weight, AGA with overweight/obesity, and LGA with overweight/obesity) demonstrated a significant increase in the odds for HBP, compared to the group with the combination of AGA and normal weight in adolescence. The combinations of HBW with overweight/obesity and being LGA with overweight/obesity were associated with an elevated BP at significantly higher aORs (aOR 4.36 and aOR 5.03, respectively) than other combinations of BW and adolescent BMI with either of the risk factors alone (HBW, LGA, or overweight/obesity) were.

**Table 4:** Associations between birth weight with BMI in adolescence and high blood pressure (univariate and multivariate analyses)

Variables	OR (95% CI) *P* value	aOR^1^ (95% CI) *P* value
Birth weight (g) and BMI categories		
BW 2500–4000 and normal weight	1.00	1.00
BW >4000 and normal weight	1.52 (1.23–1.87) *P* < 0.001	1.37 (1.11–1.70) *P* = 0.004
BW 2500–4000 and overweight/obesity	3.56 (2.95–4.31) *P* < 0.001	3.63 (2.99–4.41) *P* < 0.001
BW >4000 and overweight/obesity	4.69 (3.30–6.67) *P* < 0.001	4.36 (3.04–6.26) *P* < 0.001
Birth weight for gestational age and BMI categories		
AGA and normal weight	1.00	1.00
LGA and normal weight	1.39 (1.09–1.77) *P* = 0.008	1.40 (1.10–1.80) *P* = 0.007
AGA and overweight/obesity	3.36 (2.78–4.06) *P* < 0.001	3.39 (2.79–4.13) *P* < 0.001
LGA and overweight/obesity	4.96 (3.32–7.41) *P* < 0.001	5.03 (3.33–7.60) *P* < 0.001

*OR* crude odds ratio, *aOR* ^*1*^ adjusted odds ratios for age and sex, *CI* confidence interval, *BW* birth weight, *BMI* body mass index, *AGA* appropriate for gestational age, *LGA* large for gestational age

## Discussion

To our knowledge, this is the first report that investigated the associations between HBW and being LGA and the combinations of HBW and being LGA with BMI in adolescence and elevated BP among Lithuanian adolescents aged 12–15 years. In our study, the prevalence of HBW and being LGA were 13.9 and 10.4% respectively. In this cross-sectional study, we found that HBW (>4000 g) and being LGA were associated with a HBP among adolescents. In addition, we found that the subjects with both risk factors in combinations (HBW with overweight/obesity in adolescence and being LGA with overweight/obesity in adolescence) had higher odds for HBP, compared to subjects who had combinations with either of these risk factors alone.

Previous studies on HBW and HBP have reported different results. The comparison of the results between different studies is complicated and difficult because there are differences in epidemiological study designs, sample size, the age of the investigated participants, and the cutoff criteria for defining overweight and obesity. Researchers also apply different methods of obtaining BW data (examination of medical records, questionnaire, and interviews with parents), different number of BP measurements, various BW categories and different BW reference categories, different classifications of birth weight for gestational age. In addition, in data analysis, preterm and full-term infants are combined into a single group or divided into different groups, and different potential confounders are used. Nevertheless, our findings could confirm those of other studies that demonstrated a significant association between HBW and HBP. A cross-sectional study of Brazilian adolescents aged 11–18 years showed that the HBW group (≥4000 g), as compared with the normal BW group (>2500 and <4000 g), had significantly higher prevalence ratios for cardiovascular risk factors: high SBP—3.26, high DBP—2.99, obesity—2.63, and the metabolic syndrome—3.12 [11]. The results from the study involving 17-year-old subjects born in Jerusalem demonstrated that women with BW 4000–4499 g had a significant by 2.6-fold higher risk of high SBP compared to those with the birth weight of 3000–3499 g [26]. In the population-based study of Chinese children aged 3–6 years, children of higher BW percentiles were found to have a significantly increased risk of hypertension in both boys and girls, comparing to the lowest quartile of the BW percentile [14]. A population-based case–control study in the USA [15] found that BW ≥4000 g and being LGA were significantly associated with primary hypertension in adolescents and young adults 15–24 years of age, but not in children 8–14 years of age. However, in contrast to our study, several studies have found no significant associations between HBW and HBP in children in multivariate analyses after adjustment for confounding factors including current childhood BMI [12, 13].

Genetic and environmental factors can affect the relationships between BW and cardiometabolic risk [27]. The mechanism underlying the association between HBW and HBP is not well known. However, studies have suggested that HBW (>4000 g) is associated with an increased risk of obesity in later life [28], while overweight and obesity are significantly associated with hypertension [29]. Obesity is associated with an increased activity of the sympathetic nervous system, the activation of the renin–angiotensin system, hormonal perturbations, and renal structural damage, which can lead to hypertension [30]. It has also been demonstrated that individuals who were born with a higher than average BW had a higher risk of developing the metabolic syndrome in childhood and adolescence [31]. Moreover, HBW (≥4000 g) significantly correlates with the metabolic syndrome in childhood [32].

The present study has several limitations. Our study analyzed only a sample of 12–15-year-old adolescents of the second largest city of Lithuania. In the present study, BP readings were obtained by an automatic oscillometric BP monitor, although, according to the Fourth Report, HBP readings obtained with an oscillometric device should be repeated by using auscultation [21]. The categories of overweight and obesity were placed into a single category (overweight/obesity) due to the small number of the study participants in the overweight/obesity group. Furthermore, we did not analyze intra- or inter-observer errors in BP and anthropometric measurements in our research. In addition, as our study was a cross-sectional study in its design, we cannot establish a cause–effect relationship. Bias (selection, information, and confounding) can affect the results in observational research [33]. Another limitation is that we calculated the ORs (obtained by logistic regression) in our cross-sectional study, although some researchers suggest using prevalence ratios (obtained by Poisson regression) in the analysis of cross-sectional data, as the OR can overestimate the prevalence ratio [34]. In the present study, there was no adjustment for family history of hypertension, pubertal status, or other potential confounding factors because information on these risk factors was lacking. Detailed information regarding nutritional status or the dietary pattern was not available either in our study. Furthermore, biochemical parameters were not assessed in our research.

Despite the above-mentioned limitations, the present study confirmed a significant association of HBW and being LGA with HBP among adolescents aged 12–15 years. HBW, LGA subjects who became overweight/obese in adolescence had higher odds for HBP than those with the combinations of either of these risk factors alone did. In Lithuania, public health strategies should focus more on preventing HBW and LGA cases as well as overweight/obesity in childhood and adolescence. Early interventions to reduce cardiovascular risk factors during infancy, childhood, and adolescence can reduce the risk of cardiovascular disease. The promotion of healthy lifestyles, healthy nutrition, physical activities, and other healthy behaviors for preventing and controlling overweight, obesity, and HBP is highly important.

## Conclusions

HBW and being LGA were significantly associated with HBP among Lithuanian adolescents. The participants with HBW or those LGA at birth and with overweight/obesity in adolescence had higher odds of HBP, compared to those with other combinations of either of the risk factors alone.

These findings would be useful in the development of clinical and public health strategies for reducing the risk factors of cardiovascular diseases, and would also be important for the identification, assessment, observation, prevention, management, and treatment of HBP among adolescents. The management of cardiovascular disease risk factors should remain one of the main priorities in the development and implementation of public health strategies.

## Abbreviations

AGA: Appropriate for gestational age
aOR: Adjusted odds ratio
BMI: Body mass index
BP: Blood pressure
BW: Birth weight
CI: Confidence interval
DBP: Diastolic blood pressure
HBP: High blood pressure
HBW: High birth weight
LGA: Large for gestational age
NBP: Normal blood pressure
OR: Odds ratio
SBP: Systolic blood pressure
SD: Standard deviation
SGA: Small for gestational age
WC: Waist circumference
